# Harnessing the therapeutic value of Tanshinone IIA: a breakthrough therapy in cardiovascular diseases

**DOI:** 10.3389/fphar.2025.1620152

**Published:** 2025-07-04

**Authors:** Lanxiang Du, Chengyan Guan, Hao Zhang, Haoyue Jia, Qiang Wan

**Affiliations:** ^1^ Department of Traumatic Orthopedics, Ganzhou Traditional Chinese Medicine Hospital Affiliated to Jiangxi University of Chinese Medicine, Ganzhou, China; ^2^ Clinical Medical College, Jiangxi University of Chinese Medicine, Nanchang, China; ^3^ Department of Medical Cardiology, Affiliated Hospital of Jiangxi University of Chinese Medicine, Nanchang, China

**Keywords:** Tanshinone IIA, cardiovascular diseases, therapeutic mechanisms, molecular targets, clinical use

## Abstract

Cardiovascular diseases (CVDs) have emerged as one of the leading causes of global mortality and health burden, with their prevalence and mortality rates demonstrating a persistent upward trend, thereby posing significant challenges to public health worldwide. Tanshinone IIA (Tan IIA), the principal lipophilic bioactive component isolated from *Salvia miltiorrhiza Bunge*, has gained substantial recognition in cardiovascular therapeutics. Accumulating evidence from recent investigations has demonstrated that Tan IIA exhibits multi-target pharmacological properties and modulates diverse signaling pathways in cardiovascular protection, positioning it as a promising candidate in natural product-based drug discovery. The therapeutic efficacy is mediated through multiple mechanisms, including but not limited to anti-atherosclerotic effects, lipid homeostasis regulation, anti-arrhythmic properties, myocardial functional enhancement, and hemodynamic stabilization. This comprehensive review systematically elucidates the molecular mechanisms and therapeutic targets underlying Tan IIA’s cardio-protective effects, particularly focusing on its anti-inflammatory, antioxidant, anti-atherosclerotic, and myocardial preservation properties. Furthermore, we critically evaluate its current clinical applications and propose potential directions for future research to optimize its therapeutic potential in cardiovascular medicine.

## 1 Introduction

Cardiovascular diseases (CVDs) represent a predominant cause of global morbidity and mortality, characterized by intricate pathological mechanisms involving multiple interrelated factors, including chronic inflammation, oxidative stress, atherosclerosis, myocardial fibrosis, and ventricular remodeling (Greco et al., 2025; Ueyama et al., 2025). Despite significant advancements in modern medicine, including the development of innovative anti-platelet therapies, sophisticated vascular interventional techniques, and targeted pharmacological agents, critical challenges persist in cardiovascular management. These challenges encompass high recurrence rates, irreversible myocardial damage, and adverse drug effects. For example, a particularly pressing concern is the development of chronic heart failure secondary to post-myocardial infarction ventricular remodeling, which remains a formidable clinical challenge. Consequently, investigating natural compounds with multi-target therapeutic effects and favorable safety profiles has emerged as a pivotal research focus in cardiovascular medicine.

Tanshinone IIA (Tan IIA), the principal bioactive lipophilic constituent extracted from *Salvia miltiorrhiza Bunge*, exhibits a broad spectrum of pharmacological properties encompassing anti-inflammatory, antioxidant, anti-atherosclerotic, and cardioprotective activities (Chen, 2024; Hu K. B. et al., 2023). Recent advancements in elucidating Tan IIA’s molecular mechanisms have significantly enhanced its therapeutic potential in CVDs management, garnering considerable attention in pharmacological research. Mechanistic studies have revealed that Tan IIA not only modulates inflammatory cytokine cascades but also potentiates endogenous antioxidant systems and attenuates myocardial ischemia-reperfusion injury (Peng et al., 2023; Wu X. et al., 2023). Furthermore, emerging evidence highlights its regulatory capacity in microRNA expression profiles and inhibitory effects on myocardial fibrotic processes, underscoring its unique multi-target therapeutic characteristics (Li S. et al., 2023; Qian et al., 2023). This comprehensive review systematically examines the molecular mechanisms underlying Tan IIA’s cardiovascular protective effects, with the dual objectives of delineating its precise pharmacological targets and expanding the therapeutic horizons of traditional Chinese medicine in cardiovascular therapeutics.

## 2 Essential characteristics of Tan IIA

**FIGURE 1:**
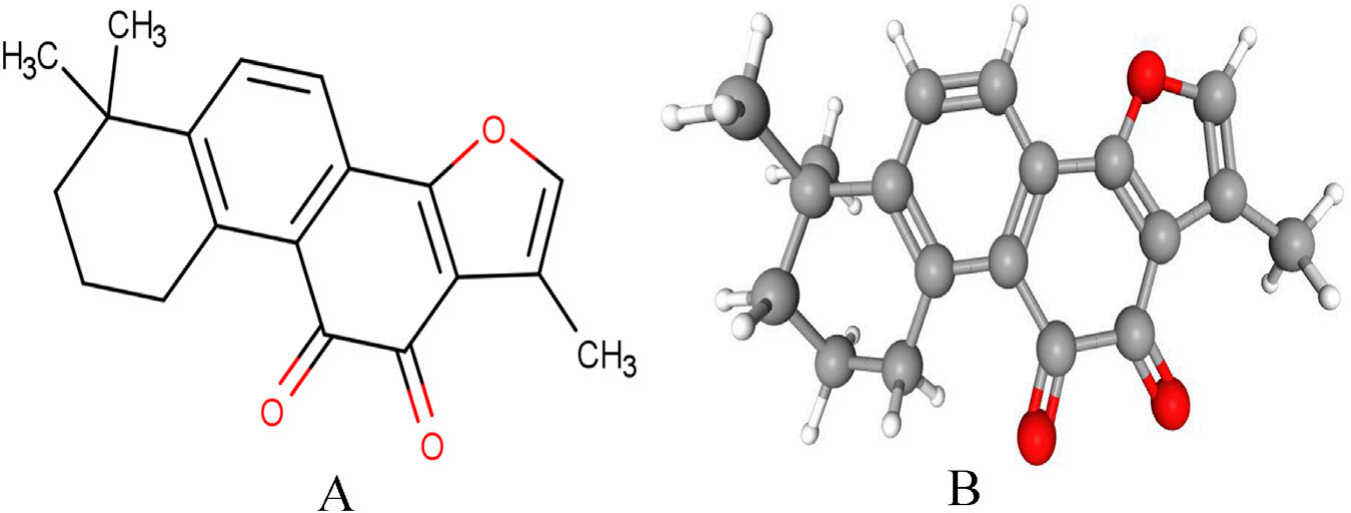
Chemical structure of Tan IIA. **(A)** Plane chemical structure of Tan IIA. **(B)** Stereochemical chemical structure of Tan IIA.

The precise timeline for the initial synthesis of Tan IIA remains unclear in the scientific literature. The structural characterization of Tan IIA (PubChem CID: 164676, chemical structure illustrated in Figure 1) was first established by Kakisawa in the 1960s (Lee et al., 1987), marking the commencement of extensive research into its synthesis and structural analogs. However, the inherent lipophilic nature of Tan IIA presents significant pharmaceutical challenges, particularly its poor aqueous solubility and consequent low bioavailability, which substantially impeded its formulation development and clinical implementation. Pharmacokinetic studies have identified two primary contributing factors to its limited oral bioavailability: P-glycoprotein-mediated intestinal efflux and extensive first-pass hepatic metabolism (Yu et al., 2007). To address these limitations, including suboptimal intestinal absorption and delayed clinical onset, structural modifications have been pursued by medicinal chemists. A significant breakthrough was achieved in 1978 when Qian et al. developed a semi-synthetic sulfonated derivative of Tan IIA, converting it into a sodium salt form. This modification markedly enhanced its aqueous solubility, enabling its formulation as an injectable preparation for intravenous administration in cardiovascular and cerebrovascular disease management. This advancement represented a pivotal milestone in Tan IIA’s clinical translation. Despite its demonstrated multi-target therapeutic potential, Tan IIA’s clinical application continues to face pharmacological challenges, particularly regarding its bioavailability and short half-life. The sulfonated derivative, while addressing solubility issues, introduced new limitations due to its excessive hydrophilicity, resulting in rapid renal clearance and incomplete therapeutic utilization. Consequently, the optimization of Tan IIA’s solubility profile while maintaining its therapeutic efficacy remains an active area of pharmaceutical research and development.

Recent advancements in pharmaceutical technology have revolutionized Tan IIA delivery strategies, primarily through structural modification and innovative nano-delivery systems. Yan et al. demonstrated that porous silica-based solid dispersions significantly enhance Tan IIA’s oral bioavailability by optimizing dissolution kinetics while ensuring formulation stability (Yan et al., 2015). Parallel research by Liu et al. revealed that low-molecular-weight chitosan solid dispersions offer a promising approach to improve dissolution rates and oral bioavailability in rats (Liu et al., 2013). In a notable advancement, Luo et al. engineered a chitosan/montmorillonite composite microsphere system, leveraging its unique interlayer architecture to achieve a superior loading capacities and sustained-release properties (Luo et al., 2019). Lipid-based nanocarriers have emerged as a particularly promising strategy. Ashour et al. developed sophisticated lipid nanocapsules that utilize biocompatible lipid matrices to enhance Tan IIA’s oral bioavailability through efficient drug encapsulation and controlled release mechanisms (Ashour et al., 2020). Similarly, Zhu et al. achieved remarkable progress in Tan IIA mesoporous silica nanoparticles functionalized with polyethyleneimine-polyethylene glycol, demonstrating optimal dispersibility, particle size distribution, and sustained release characteristics (Zhu et al., 2021). Innovative delivery systems have been developed for specific therapeutic applications. Ye et al. engineered an injectable borneol-modified Tan IIA liposomal formulation, demonstrating enhanced bioavailability and superior brain tissue penetration compared to conventional Tan IIA sulfonate preparations (Wang et al., 2020b). Furthermore, transdermal delivery has been advanced through nanocrystal-porous silica composite cataplasms, showing excellent skin permeability in rats (Gu et al., 2021). Microemulsion technology has also shown promise, with Ma et al. developing a Tan IIA-loaded microemulsion that effectively circumvents first-pass metabolism (Ma et al., 2022). Targeting specific pathological conditions, Zhang et al. designed discoidal reconstituted high-density lipoproteins as precision nanocarriers for atherosclerotic plaque targeting (Zhang et al., 2011). Zhan et al. fabricated a self-dissolving microneedle loaded with Tan IIA, which was shown to effectively suppress human skin fibroblast proliferation (Zhan et al., 2023). Besides, cutting-edge biomaterials have further expanded Tan IIA’s therapeutic potential. Fan et al. pioneered an injectable liquid metal-sodium alginate composite, achieving stable drug release and demonstrating efficacy in intrapericardial administration for CVDs (Fan et al., 2024). While these technological breakthroughs have significantly addressed solubility and absorption limitations, ongoing research efforts remain crucial to develop more sophisticated dosage forms that can further optimize Tan IIA’s bioavailability and therapeutic efficacy in clinical settings.

Tan IIA possesses a fused aromatic ring system with diketone groups, conferring high lipophilicity that facilitates its passive diffusion across cell membranes. This property enables intracellular accumulation and interaction with hydrophobic targets, including mitochondria and nuclear receptors, underpinning its anti-inflammatory, antioxidant, and anti-apoptotic effects (Yang et al., 2023). The compound’s tissue distribution profile like preferential accumulation in lipid-rich organs such as adipose tissue, liver, and brain, contributes to its therapeutic efficacy in CVDs (Guo et al., 2020). Furthermore, its stability in lipid-rich environments prolongs its pharmacological action, ensuring sustained target inhibition. Structurally, Tan IIA’s planar quinone core restricts conformational flexibility but enhances binding affinity to protein kinase domains through hydrophobic interactions. This rigidity enables selective binding to key proteins, such as those involved in angiogenesis, and modulates L-type calcium channels to suppress Ca^2+^ influx, thereby exerting anti-arrhythmic and vasodilatory effects (Ansari et al., 2021). The pharmacological mechanisms of Tan IIA are intrinsically linked to its physicochemical properties such as lipophilicity, redox activity, and structural rigidity, which collectively govern its cellular permeability, target-binding specificity, metabolic stability, and antioxidant/anti-inflammatory activities. Optimizing these characteristics through structural modifications or advanced formulation strategies remains pivotal for enhancing its clinical efficacy.

## 3 The clinical use of Tan IIA in the treatment of CVDs

Clinical evidence has demonstrated that both Tanshinone IIA (Tan IIA) and its water-soluble derivative sodium Tanshinone IIA sulfonate (STS) exhibit significant therapeutic benefits in CVDs. In a multicenter, randomized controlled trial involving 372 patients with non-ST elevation acute coronary syndrome undergoing percutaneous coronary intervention (PCI), STS administration significantly reduced peri-procedural myocardial injury and decreased the incidence of short-term major adverse cardiac events (MACEs) (Mao et al., 2021). A clinical study of 300 acute myocardial infarction patients post-PCI demonstrated that Tan IIA injection effectively improved coronary microcirculation, attenuated ventricular remodeling, enhanced cardiac function, and reduced MACE occurrence (Lu et al., 2021). In addition, a prospective randomized trial with 101 ST-elevated myocardial infarction patients showed that STS treatment markedly prevented adverse left ventricular remodeling and limited neutrophil-mediated myocardial damage in the infarct zone (Mao et al., 2019). Besides, another randomized controlled trial comprising 72 participants with either unstable angina or non-ST-elevation myocardial infarction revealed that STS therapy significantly improved cardiac function while reducing inflammatory markers, including high-sensitivity C-reactive protein (hs-CRP), interleukin-6 (IL-6), monocyte chemotactic protein-1 (MCP-1), and soluble CD40 ligand (sCD40L) (Li et al., 2017). These well-designed clinical studies collectively demonstrate that Tan IIA represents a safe and effective therapeutic option for various cardiovascular conditions. However, further large-scale clinical investigations are warranted to fully elucidate its therapeutic potential and optimize clinical applications.

## 4 Mechanism and target of Tan IIA in treating CVDs

### 4.1 Atherosclerosis

Atherosclerosis, a prevalent and pathologically complex cardiovascular disorder, is characterized by the progressive accumulation of lipid deposits and fibroproliferative changes within the arterial wall, leading to luminal stenosis, vascular stiffening, and compromised hemodynamic function (Libby, 2021). This chronic inflammatory disease process involves the formation of atheromatous plaques, which are complex structures comprising lipid cores, vascular smooth muscle cells (VSMCs), and inflammatory infiltrates including macrophages and lymphocytes within the arterial intima (Certo et al., 2024). The pathophysiological progression of atherosclerosis typically initiates with endothelial dysfunction, followed by the development of fatty streaks that evolve into advanced plaques, ultimately resulting in significant luminal obstruction or complete vascular occlusion. The distribution of atherosclerotic lesions predominantly affects large and medium-sized arteries, with a particular predilection for coronary, cerebrovascular, and peripheral arterial systems. The course of atherosclerotic plaques involves progressive calcification and vascular remodeling, contributing to arterial rigidity and impaired vascular compliance. Of particular clinical significance is the phenomenon of plaque vulnerability, characterized by thin fibrous caps, large lipid cores, and intense inflammatory activity, which predisposes to plaque rupture and subsequent thromboembolic complications. In recent years, Tan IIA has emerged as a promising therapeutic agent in atherosclerosis management.

#### 4.1.1 Suppressing VSMCs proliferation and migration

VSMCs play a pivotal role in the pathogenesis of atherosclerosis, contributing significantly to arterial stiffening and luminal narrowing. This complex pathological process is mediated through multiple interconnected biological mechanisms and signaling pathways. The proliferative activity of VSMCs results in cellular hyperplasia, leading to fibrous cap formation that critically influences the stability of atherosclerotic plaques. Furthermore, migratory VSMCs not only augment the cellular density within the intimal layer but also engage in intricate interactions with infiltrating immune cells, thereby contributing to the development of more sophisticated plaque architecture. The cellular aggregation within the plaque microenvironment facilitates lipid deposition and perpetuates inflammatory cascades. The proliferation and migration of VSMCs represent dynamic and interrelated processes, wherein proliferating VSMCs can maintain their proliferative capacity in novel microenvironments, while migratory cells possess the potential to establish new cell populations within the intima (Yu et al., 2024). This reciprocal cellular behavior profoundly influences the morphological evolution and functional characteristics of atherosclerotic plaques. Specific growth factors, particularly transforming growth factor-β (TGF-β) and platelet-derived growth factor (PDGF), exert their biological effects through receptor-mediated activation of intracellular signaling cascades, thereby orchestrating VSMC proliferation and migration (Wang et al., 2022). These molecular mediators are typically upregulated in response to vascular endothelial injury and subsequent inflammatory processes.

Tan IIA has demonstrated significant efficacy in mitigating atherosclerotic lesions in mice through the inhibition of VSMC proliferation and migration (Wang et al., 2017). Emerging evidence has established microRNAs (miRs) as crucial regulatory molecules in the pathogenesis of atherosclerosis. Mechanistic study revealed that Tan IIA exerted its anti-atherosclerotic effects by suppressing VSMCs proliferation through down-regulating miR-712-5p (Qin et al., 2020). Furthermore, Li et al. demonstrated that Tan IIA effectively inhibits oxidized-low density lipoprotein (ox-LDL)-induced VSMC proliferation and migration via modulation of the miR-137/TRPC3 axis (Li W. et al., 2023). In a separate investigation, Li et al. elucidated that Tan IIA attenuated homocysteine-induced VSMCs proliferation through regulation of the miR-145/CD40 pathway (Li et al., 2020). The therapeutic potential of Tan IIA extends to metabolic stress conditions, as evidenced by its ability to ameliorate high glucose-induced VSMCs proliferation and migration via downregulation of miR-21-5p and tropomyosin 1 expression (Jia et al., 2019). Lou et al. provided additional mechanistic insight, demonstrating that Tan IIA suppresses VSMCs proliferation and migration by up-regulating Krüppel-like factor 4 (KLF4) expression and mediating VSMC phenotypic modulation in rat models (Lou et al., 2020). The pharmacological effects of Tan IIA also encompass the regulation of hormonal and inflammatory pathways. Experimental study has shown that Tan IIA administration effectively suppresses angiotensin II (Ang II)-induced VSMCs proliferation and autophagy through inhibition of the p38 MAPK pathway (Lu et al., 2019). Similarly, Lu et al. reported that Tan IIA attenuates advanced glycation end products (AGEs)-induced VSMCs proliferation and migration by inhibiting the ERK1/2 signaling cascade (Lu et al., 2018). Additionally, Tan IIA inhibited TNF-α-induced VSMCs proliferation and migration via suppression of protein kinase B (Akt) phosphorylation and matrix metalloproteinase-9 (MMP-9) activity (Jin et al., 2008).

#### 4.1.2 Improving vascular endothelial dysfunction

Vascular endothelial cells (VECs), forming the innermost layer of the vascular wall, serve as critical regulators of vascular homeostasis through multiple essential functions. These include maintenance of vascular permeability, hemodynamic regulation, synthesis of vasoprotective mediators such as nitric oxide (NO), and immunomodulatory activities (Wei X. et al., 2024). Under physiological conditions, VECs play a pivotal role in maintaining vascular health by preventing inflammatory processes and thrombus formation. However, various pathological stimuli, including hypertension, hyperglycemia, dyslipidemia, tobacco exposure, and inflammatory cytokines, can induce endothelial dysfunction, thereby initiating the atherosclerotic cascade. The pathogenesis involves chemokines and cell adhesion molecules up-regulating, which serve as inflammatory mediators that recruit circulating immune cells, particularly monocytes and lymphocytes, into the vascular intima, establishing a chronic inflammatory state. Dysfunctional VECs facilitate the transendothelial migration and subsequent oxidative modification of LDL within the subendothelial space. The resulting ox-LDL not only perpetuates the inflammatory response but also induces further endothelial injury (Panduga et al., 2024). Furthermore, endothelial dysfunction exacerbates atherogenesis through the dysregulation of vasoactive substances, characterized by diminished NO bioavailability and altered endothelin-1 (ET-1) secretion, coupled with enhanced platelet activation and prothrombotic tendency.

Wei et al. demonstrated that STS attenuates human umbilical vein endothelial cells (HUVECs) injury in mice through modulation of the A20-nuclear factor kappa B (NF-κB)-NOD-like receptor family pyrin domain containing 3 (NLRP3) inflammasome-catalase (CAT) axis (Wei W. et al., 2024). In a complementary study, Wu et al. revealed that Tan IIA protects against oxidative stress-induced VECs pyroptosis in mice by regulating the thioredoxin-interacting protein (TXNIP)/NLRP3 inflammasome pathway (Wu Q. et al., 2023). Recognizing homocysteine (Hcy) as an independent atherogenic factor, Zhou et al. reported that STS mitigates Hcy-induced VECs injury through suppression of intracellular oxidative stress and mitochondrial dysfunction. This protective effect is mediated via activation of the nicotinamide N-methyltransferase (NNMT)/sirtuin 1 (SIRT1)-dependent nuclear factor erythroid 2-related factor 2 (Nrf2)/heme oxygenase-1 (HO-1) and Akt/MAPK signaling cascades (Zhou et al., 2023). Similarly, Tan IIA has been shown to protect human coronary artery endothelial cells (HCAECs) by inhibiting ferroptosis through Nrf2 pathway activation (He et al., 2021). Moreover, Tan IIA exhibits potent protective effects against oxidative stress-induced HUVECs injury, mediated through estrogen receptor (ER) and cyclic adenosine monophosphate (cAMP) signaling pathways (Yan et al., 2021). The mitochondrial pathway has been identified as another crucial mechanism of Tan IIA’s endothelial protection. Zhu et al. demonstrated that Tan IIA alleviates hydrogen peroxide (H_2_O_2_)-induced HUVECs injury through regulation of mitochondrial apoptotic pathway (Zhu H. et al., 2017). This finding was corroborated by Chan et al., who showed that Tan IIA pretreatment reduces H_2_O_2_-induced HUVECs apoptosis by attenuating oxidative stress through elevating activating transcription factor-3 (ATF-3) expression (Chan et al., 2012). Endothelial progenitor cells (EPCs), the precursor cells of VECs, play a critical role in vascular repair through their mobilization from bone marrow to peripheral circulation in response to physiological and pathological stimuli. Yang et al. demonstrated that Tan IIA exerts anti-inflammatory effects in TNF-α-stimulated EPCs by down-regulating vascular cell adhesion molecule-1 (VCAM-1) and intercellular adhesion molecule-1 (ICAM-1) expression through inhibition of the NF-κB pathway (Yang et al., 2016). Additionally, Tan IIA has been shown to enhance TNF-α-induced EPCs proliferation, migration, adhesion, and vasculogenic capacity while suppressing the release of inflammatory mediators, including MCP-1, IL-6, and sCD40L (Wang et al., 2015). Heng et al. reported that Tan IIA preserves EPCs proliferation and differentiation capacity by maintaining CAT activity through inhibition of the receptor for advanced glycation end products (RAGE)-TXNIP-NLRP3 inflammasome pathway (Heng et al., 2023). Chen et al. further elucidated that Tan IIA attenuates ox-LDL-induced VECs injury through modulation of the circ_0000231/miR-590-5p/TXNIP axis, mediated by NF-κB pathway inhibition (Chen et al., 2024). Considering the critical role of mitochondrial dysfunction in atherogenesis, Zhu et al. demonstrated that Tan IIA maintains mitochondrial homeostasis and suppresses mitochondrial reactive oxygen species (ROS) overproduction through AMP-activated protein kinase (AMPK) pathway regulation, thereby alleviating VECs pyroptosis in atherosclerotic mice (Zhu et al., 2022).

Emerging evidence indicates that angiogenesis triggered by VECs injury plays a pivotal role in the pathogenesis of atherosclerosis. Lee et al. demonstrated that Tan IIA effectively inhibits vascular endothelial growth factor (VEGF)-induced EPCs angiogenesis through suppression of the phospholipase C (PLC), Akt, and JNK pathways (Lee et al., 2017). The anti-angiogenic properties of Tan IIA are further mediated through inhibition of the VEGF/VEGF receptor 2 (VEGFR2) signaling cascade in VECs (Xing et al., 2015). Chen et al. provided mechanistic insights into the therapeutic potential of STS, showing its ability to ameliorate post-ischemic angiogenesis in mice through dual modulation of miR-133a suppression and GTP cyclohydrolase 1 (GCH1) protein upregulation (Chen L. et al., 2019). Furthermore, Tan IIA has been shown to attenuate hypoxia-induced angiogenesis by down-regulating VEGF and basic fibroblast growth factor (bFGF) expression through inhibition of hypoxia-inducible factor 1α (HIF-1α) (Sui et al., 2017). Zhao et al. reported that STS alleviates hypoxic trophoblast-induced HUVECs dysfunction through targeted inhibition of high mobility group box 1 (HMGB1) release and subsequent reduction of VCAM-1 and ICAM-1 secretion (Zhao et al., 2017). The protective effects of Tan IIA on hypoxia-induced endothelial dysfunction extend to the glucose metabolism regulating, as evidenced by its ability to modulate glucose transporter 1 (GLUT-1) expression through HIF-1α pathway activation (Zhou et al., 2022). The involvement of intracellular chloride channel 1 (CLIC1) in oxidative stress and inflammatory responses has been elucidated by Zhu et al., who demonstrated that STS ameliorates VECs dysfunction in atherosclerotic mice through CLIC1 downregulation (Zhu J. et al., 2017). Additionally, Tan IIA micelles have shown significant endothelial protective effects by inhibiting VCAM-1 secretion and inflammatory cascades through suppression of the NF-κB pathway (Liu et al., 2022). Endothelial-mesenchymal transition (EndMT), a dynamic cellular process characterized by endothelial cell transformation into mesenchymal phenotypes under pathological stimuli, has emerged as a critical mechanism in atherosclerotic progression. Recent investigation has revealed that Tan IIA attenuates bleomycin-induced EndMT by preserving VECs tube formation capacity through modulating Akt/mammalian target of rapamycin (mTOR)/p70S6K pathway (Jiang et al., 2019). This finding underscores the therapeutic potential of Tan IIA in modulating endothelial plasticity during atherogenesis.

#### 4.1.3 Inhibiting macrophage-derived foam cells formation

Foam cells, representing a crucial cellular component in atherogenesis, are characterized by the excessive intracellular accumulation of lipids, primarily cholesterol esters, within macrophages or VSMCs following the recognition and uptake of ox-LDL. The formation of these lipid-laden cells is mediated through specific surface receptors, including cluster of differentiation 36 (CD36) and scavenger receptor-A (SR-A), which facilitate the internalization of ox-LDL (Saki et al., 2024). The transformation of macrophages into foam cells is marked by the progressive accumulation of cytoplasmic lipid droplets, creating a distinctive foamy appearance. This cellular transformation represents a hallmark event in the development of atherosclerotic lesions. Foam cells contribute significantly to the chronic inflammatory milieu within the vascular wall through the sustained release of chemokines, and proteolytic enzymes.

The pathological significance of foam cells extends beyond their inflammatory role. As these cells proliferate and eventually undergo necrosis, they exacerbate local inflammation and contribute to plaque instability. Furthermore, foam cells secrete MMPs, which degrade the extracellular matrix components of the vascular wall, potentially leading to plaque rupture or calcification, these processes collectively increase the risk of thrombotic complications (Li et al., 2024). Consequently, therapeutic strategies targeting foam cell formation represent a crucial approach in the management of atherosclerosis, offering potential for plaque stabilization and prevention of disease progression.

Tan IIA has demonstrated significant anti-atherosclerotic potential in LDL receptor (LDLR) knockout mice through its ability to reduce lipid accumulation and inhibit macrophage-derived foam cell formation by elevating macrophage efferocytosis (Wang et al., 2023). Qian et al. elucidated that Tan IIA attenuates atherosclerosis progression by modulating lipid metabolism and suppressing foam cells formation through regulation of the miR-214-3p/autophagy-related protein-16-like protein 1 (ATG16L1) axis and MAPK/mTOR-mediated autophagy pathways (Qian et al., 2023). Complementing these findings, Liu et al. demonstrated that Tan IIA inhibits atherogenesis and reduces cholesterol accumulation in macrophage-derived foam cells through up-regulating ATP-binding cassette transporter A1 (ABCA1) and ABCG1 expression, mediated by activation of the ERK/Nrf2/HO-1 signaling cascade (Liu et al., 2014). Omentin-1 is a novel adipocytokine that possesses a protective role in the cardiovascular system. The therapeutic effects of Tan IIA extend to the regulation of adipocytokine signaling, as evidenced by Tan et al.'s report that Tan IIA attenuates atherosclerosis in apolipoprotein E-deficient (ApoE^−/−^) mice by promoting cholesterol efflux and alleviating lipid accumulation in macrophages through modulation of the Omentin-1/ABCA1 pathway (Tan et al., 2019). Chen et al. provided further mechanistic insights, showing that Tan IIA stabilizes vulnerable atherosclerotic plaques by reducing foam cells accumulation through inhibition of the TLR4/MyD88/NF-κB pathway (Chen Z. et al., 2019). The regulation of scavenger receptor activity represents another mechanism of Tan IIA’s effect, as demonstrated by Xu et al., who reported that Tan IIA suppresses ox-LDL uptake and inhibits plaque formation in ApoE^−/−^ mice by down-regulating lectin-like ox-LDL receptor-1 (LOX-1) expression via NF-κB pathway inhibition (Xu et al., 2012). Besides, Zhang et al. revealed additional cholesterol metabolism pathways influenced by Tan IIA, showing its ability to ameliorate atherosclerosis in rabbits by targeting foam cells formation through promotion of scavenger receptor class B type I (SR-BI) and cholesteryl ester (CE)-triglyceride (TG) interchange, along with regulation of TG-rich lipoprotein metabolism (Zhang et al., 2012). Furthermore, the role of macrophage polarization in atherogenesis has been increasingly recognized, with M1-type macrophages demonstrating impaired lipid processing capacity, leading to foam cells formation and subsequent plaque progression. Tan IIA has been shown to modulate this process through its ability to alleviate atherosclerosis in ApoE^−/−^ mice by orchestrating the crosstalk between autophagy and macrophage polarization via KLF4 activation and miR-375 suppression (Chen W. et al., 2019).

#### 4.1.4 Inhibiting inflammatory reaction

Inflammatory response plays a pivotal role in the pathogenesis and progression of atherosclerosis, as well as in the destabilization and rupture of atherosclerotic plaques. This inflammatory process not only exacerbates vascular stenosis and arterial stiffening but also dramatically contributes to both the progression and instability of atherosclerotic plaques. During the initial phases of atherosclerosis, pro-inflammatory cytokines are released, which subsequently trigger endothelial cells activation, leukocyte recruitment, and lipid accumulation (Kong et al., 2022). Additionally, chemokines such as MCP-1 facilitate the migration of monocytes into the arterial wall, amplifying the inflammatory cascade and accelerating atherosclerotic plaque formation (Bianconi et al., 2018; Sun et al., 2023). The chronic inflammatory milieu promotes the proliferation of VSMCs and the deposition of extracellular matrix components, ultimately leading to vascular fibrosis. Importantly, inflammation not only fosters plaque development but also compromises the structural integrity of the fibrous cap, rendering plaques vulnerable to rupture. Such plaque disruption can precipitate thrombotic events, resulting in acute coronary syndromes, including unstable angina. Furthermore, MMPs, particularly MMP-2 and MMP-9, contribute to plaque instability by degrading the extracellular matrix components of the vascular wall, thereby weakening the fibrous cap and increasing the propensity for plaque rupture (Liu B. et al., 2024).

Cyclooxygenase-2 (COX-2), a key enzyme in the arachidonic acid epoxidase pathway, plays a crucial role in inflammatory processes and atherosclerosis progression. Its over-expression has been implicated in promoting atherosclerotic plaque instability. Recent studies have elucidated the anti-atherosclerotic mechanisms of Tan IIA through its modulation of various inflammatory pathways. Ma et al. demonstrated that Tan IIA exerts anti-atherosclerotic effects by down-regulating COX-2 expression and mitigating endothelial inflammation through NF-κB pathway modulation (Ma et al., 2023). This finding is further supported by Meng et al., who reported that Tan IIA significantly attenuates lipopolysaccharide (LPS)-induced inflammatory responses in VSMCs via suppression of the TLR4/transforming growth factor-β-activated kinase 1 (TAK1)/NF-κB signaling axis (Meng et al., 2019). The anti-inflammatory properties of Tan IIA extend to its regulatory effects on cytokine expression. Xuan et al. observed reduced levels of inflammatory mediators in cardiac and aortic tissues of ApoE^−/−^ mice following Tan IIA treatment, mediated through downregulation of miR-146b and miR-155 (Xuan et al., 2017). Similarly, Yang et al. demonstrated that Tan IIA alleviates ox-LDL-induced inflammatory responses in macrophages by suppressing mRNA-33 and pro-inflammatory cytokines (Yang et al., 2019). The molecular mechanisms underlying Tan IIA’s protective effects involve multiple signaling pathways. Studies have identified its action through the TGF-β/phosphatidylinositol 3-kinase (PI3K)/Akt/endothelial nitric oxide synthase (eNOS) pathway (Wang et al., 2020c), and its regulation of wingless-type MMTV integration site family member 5a (Wnt5a)-mediated inflammation (Awan et al., 2022). Yuan et al. specifically reported that Tan IIA inhibits adipogenesis and inflammatory responses in ox-LDL-induced macrophages by modulating the miR-130b/Wnt5a axis (Yuan et al., 2020). Tan IIA also demonstrates significant effects on cellular interactions in atherosclerosis. It inhibits monocyte adhesion to VECs by downregulating VCAM-1, ICAM-1, and CX3CL1 expression (Chang et al., 2014), and modulates platelet-derived amyloid β peptide (Aβ) secretion through PI3K/Akt pathway activation (Shi et al., 2014). Furthermore, it regulates dendritic cell function by reducing CD86 and CD54 expression while inhibiting IL-1 and IL-12 release (Li et al., 2014). Recent advances in transcriptomic analysis have provided deeper insights into Tan IIA’s mechanisms. Chen et al. employed sequencing technology to identify non-coding RNA expression patterns in atherosclerotic lesions of Tan IIA-treated ApoE^−/−^ mice. Their findings revealed the involvement of multiple signaling pathways, including Ras, Rap1, MAPK, cAMP, and T cell receptor pathways. The competitive endogenous RNA network analysis identified key anti-inflammatory nodes: circ-Tns3/let-7d-5p/Ctsl, circ-Wdr91/miR-378a-5p/Msr1, and circ-Cd84/miR-30c/Tlr2 (Chen et al., 2020).

The NLRP3 inflammasome plays a pivotal role in mediating immune responses and contributing to the pathogenesis of various inflammatory diseases. This multiprotein complex can be activated by diverse pathogen-associated molecular patterns (PAMPs) and damage-associated molecular patterns (DAMPs), leading to caspase-1 activation and subsequent maturation and release of pro-inflammatory cytokines, these molecular events trigger inflammatory cascades that significantly contribute to atherosclerosis (Yang et al., 2024). Given its central role in inflammatory processes, the NLRP3 inflammasome has emerged as a promising therapeutic target for atherosclerosis intervention. Recent studies have demonstrated the therapeutic potential of Tan IIA in modulating NLRP3 inflammasome activity. Experimental evidence shows that Tan IIA attenuates atherosclerotic progression in ApoE^−/−^ mice through multiple mechanisms: suppression of NLRP3 inflammasome activation, downregulation of LOX-1 and CD36 expression, and amelioration of mitochondrial and lysosomal dysfunction (Wen et al., 2020). Similarly, STS has been shown to exert anti-atherosclerotic effects in ApoE^−/−^ mice by reducing MMP-2 and MMP-9 expression, inhibiting spleen tyrosine kinase phosphorylation, and suppressing NLRP3 inflammasome activation (Liu H. H. et al., 2024). Further mechanistic insights into Tan IIA’s anti-inflammatory properties were provided by Liu et al., who demonstrated that Tan IIA ameliorates LPS-induced inflammatory responses in mice through a unique mechanism involving succinate dehydrogenase inactivation in macrophages, mediated by NLRP3 inflammasome inhibition (Liu et al., 2021). These findings collectively highlight the therapeutic potential of targeting the NLRP3 inflammasome in atherosclerosis management.

#### 4.1.5 Suppressing oxidative stress damage

Oxidative stress represents a pathophysiological state characterized by an imbalance between the generation and elimination of ROS, resulting in the accumulation of these reactive molecules and subsequent cytotoxic effects. This imbalance may arise from either excessive endogenous production of oxygen free radicals or increased intake of exogenous oxidants. In the context of atherosclerosis, oxidative stress primarily contributes to disease pathogenesis through three distinct mechanisms: oxidative modification of cellular components, promotion of cellular proliferation, and modulation of vascular gene expression (Dos Santos et al., 2022). At the vascular level, oxidative stress induces the oxidative modification of ox-LDL, activates VSMCs and macrophages, and alters the expression of key adhesion molecules and chemokines, including VCAM-1 and MCP-1. Notably, emerging evidence suggests a dual role of oxidative stress in atherosclerosis development. While severe oxidative stress promotes atherogenesis, mild oxidative stress appears to play a protective role by physiologically regulating vascular gene expression and inducing the expression of anti-atherosclerotic genes, thereby maintaining vascular homeostasis (Shao et al., 2024). This paradoxical relationship between oxidative stress and atherosclerosis highlights the complexity of redox biology in vascular pathophysiology. The intensity and duration of oxidative stress appear to be critical determinants of its biological effects, with low-level oxidative stress potentially serving as an adaptive mechanism, while excessive oxidative stress contributes to vascular damage and disease progression.

**FIGURE 2:**
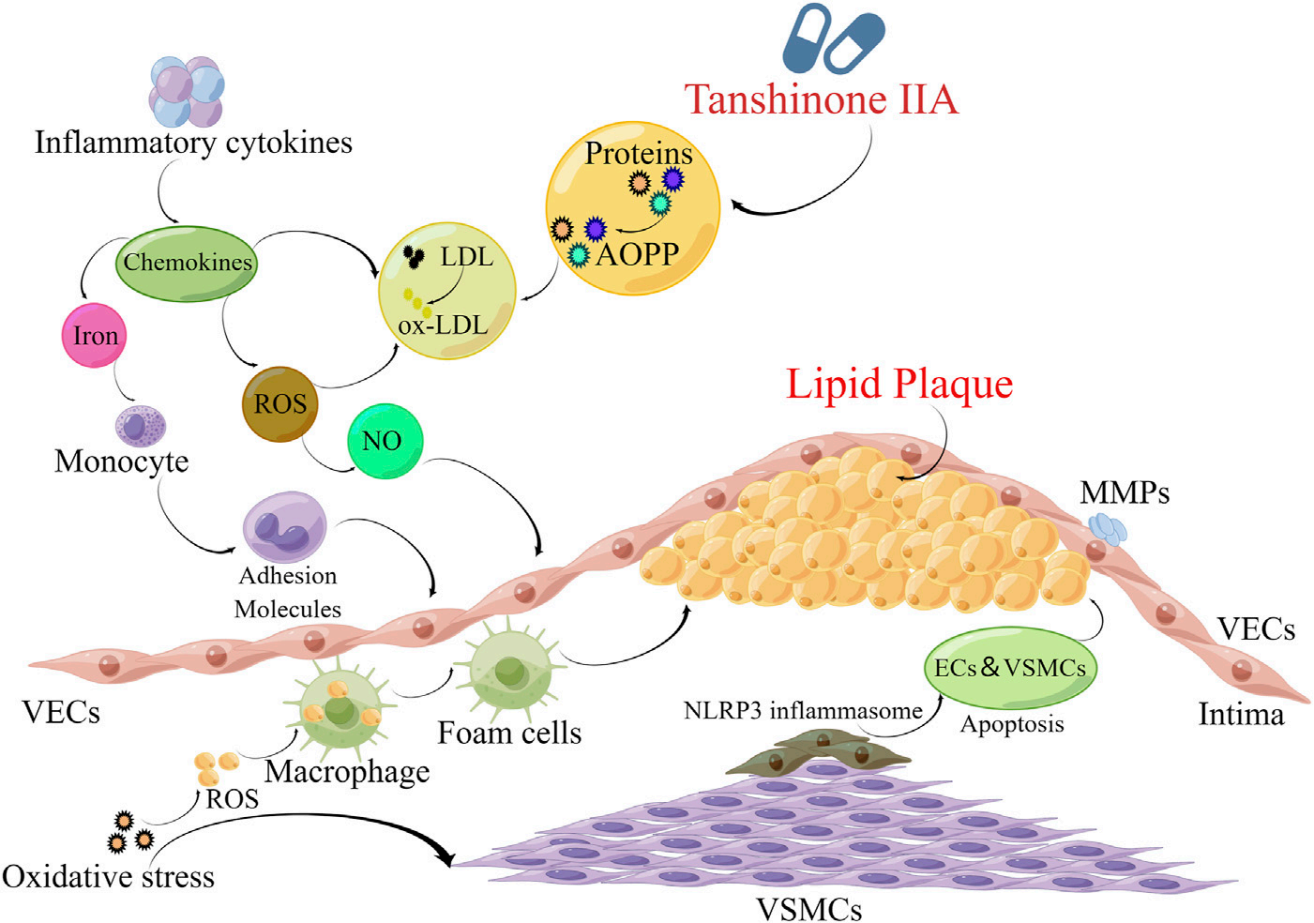
Anti-atherosclerotic effects of Tan IIA.

Nicotinamide adenine dinucleotide phosphate (NADPH) oxidase, a critical enzyme complex involved in multifarious physiological processes including cellular pathway and host defense, plays a pivotal role in redox biology by catalyzing the production of ROS from NADPH. These ROS molecules serve as important mediators in immune responses, apoptotic processes, and signal transduction pathways. Recent studies have extensively investigated the antioxidative properties of Tan IIA and its derivatives in the context of atherosclerosis. The antioxidative mechanisms of Tan IIA have been demonstrated through multiple experimental models. In *porphyromonas gingivalis*-induced atherosclerosis, Tan IIA ameliorates disease progression by reducing NOX2 and NOX4 expression, decreasing ROS production, and attenuating oxidative stress through NF-κB pathway downregulation (Xuan et al., 2023). Chen et al. further elucidated that Tan IIA inhibits atherosclerotic lesion formation in hyperlipidemic rabbits by reducing ox-LDL generation while enhancing the activities of key antioxidant enzymes, superoxide dismutase (SOD) and glutathione peroxidase (GSH-Px) (Chen et al., 2012). The therapeutic potential of STS was demonstrated by Zhu et al., who reported its ability to reduce ROS and malondialdehyde (MDA) levels in atherosclerotic mice through CLIC1 downregulation (Zhu J. et al., 2017). Similarly, Fang et al. observed that Tan IIA mitigates atherogenesis in rabbits by modulating oxidative stress markers, including decreased MDA levels and CD40 expression, reduced MMP-2 activity, and increased SOD activity (Fang et al., 2008). Moreover, the antioxidative effects of Tan IIA extend to its interaction with nuclear receptors. Pregnane X receptor (PXR) is a member of the nuclear receptor superfamily, and its activation has shown significant effects in anti-oxidative stress and protection of cardiovascular health (Shen et al., 2024). Zhu et al. demonstrated that Tan IIA protects HUVECs against H_2_O_2_-induced oxidative stress by enhancing GSH-Px activity through PXR activation (Zhu H. et al., 2017). This finding is complemented by Jia et al.'s observation that Tan IIA reduces endothelial cells apoptosis by decreasing MDA and ROS levels in oxidative stress conditions (Jia et al., 2012). At the molecular level, Tan IIA exhibits unique mechanisms in modulating oxidative stress-related pathways. It alleviates oxidative damage and inhibits platelet activation by down-regulating CD36 expression through suppression of the MAPK kinase 4 (MKK4)/JNK2 signaling pathway (Wang H. et al., 2020). Furthermore, in ovariectomized ApoE^−/−^ mice, Tan IIA demonstrates ER-mediated antioxidative effects by elevating serum SOD levels and suppressing ERK pathway activation (Liu et al., 2015). The comprehensive antioxidative properties of Tan IIA are further evidenced by its ability to attenuate atherosclerotic calcification and regulate vascular lipid and calcium homeostasis through protection against superoxide anion-induced LDL oxidation (Tang et al., 2007). These multifaceted mechanisms contribute to Tan IIA’s overall anti-atherosclerotic effects, as generalized in Figure 2 and Table 1.

**TABLE 1:** The anti-atherosclerotic molecular mechanisms of Tan IIA.

Animals or cells	Range of dosage	The specifific molecular mechanisms	Ref.
Mice	30 mg/kg	Inhibited VSMCs proliferation and migration	Wang et al. (2017)
Mice	10 mg/kg	Downregulated miR-712-5p	Qin et al. (2020)
VSMCs	2.5–10 μg/mL	Regulated miR-137/TRPC3 axis	Li et al. (2023b)
VSMCs	1–10 μM	Regulated miR-145/CD40 signaling pathway	Li et al. (2020)
Human VSMCs	10 μM	Downregulated miR-21-5p and tropomyosin 1	Jia et al. (2019)
Rats	5 mg/kg	Promoted KLF4 expression, mediated VSMCs phenotypic transformation	Lou et al. (2020)
VSMCs	5 μg/mL	Downregulated p38 MAPK pathway	Lu et al. (2019)
VSMCs	10 μM	Suppressed ERK1/2 pathway	Lu et al. (2018)
VSMCs	0–100 μM	Suppressed Akt phosphorylation and MMP-9 activity	Jin et al. (2008)
Mice	10 mg/kg	Alleviated VECs injury, regulated A20-NF-κB-NLRP3 inflammasome-CAT pathway	Wei et al. (2024a)
Mice	10 mg/kg	Alleviated oxidative stress induced-VECs pyroptosis, modulated TXNIP/NLRP3 inflammasome	Wu et al. (2023a)
HUVECs	25–100 μM	Activated NNMT/SIRT1-mediated NRF2/HO-1 and Akt/MAPKs pathways, suppressing intracellular oxidative stress and mitochondria dysfunction	Zhou et al. (2023)
HCAECs	50 μM	Activated Nrf2 pathway, inhibited ferroptosis	He et al. (2021)
HUVECs	0–40 μg/mL	Regulated ER and cAMP pathway	Yan et al. (2021)
HUVECs	5–20 μM	Activated PXR, modulated mitochondrial apoptosis pathway	Zhu et al. (2017a)
HUVECs	3–10 μM	Relieved oxidative stress damage, activated ATF-3 expression	Chan et al. (2012)
EPCs	0–20 μM	Inhibited VCAM-1 and ICAM-1 expression, blocked NF-κB pathway	Yang et al. (2016)
EPCs	0–20 μM	Inhibited MCP-1, IL-6 and sCD40L secretion	Wang et al. (2015)
Mice	10 mg/kg	Inhibited RAGE-TXNIP-NLRP3 inflammasome pathway, suppressed EPCs senescence	Heng et al. (2023)
HUVECs	5 μM	Modulated circ_0000231/miR-590-5p/TXNIP axis, blocked NF-κB pathway	Chen et al. (2024)
Mice	10 mg/kg	Alleviated VECs mitochondrial injury and pyroptosis, regulated AMPK pathway	Zhu et al. (2022)
EPCs	1–10 μM	Inhibited VEGF-induced EPCs angiogenesis, suppressed PLC, Akt and JNK pathways	Lee et al. (2017)
HUVECs	4–12 μM	Inhibited HUVECs angiogenesis, suppressed VEGF/VEGFR2 pathway	Xing et al. (2015)
Mice	10 mg/kg	Ameliorate post-ischemic angiogenesis, suppressed miR-133a, elevated GCH1 protein	Chen et al. (2019a)
HUVECs	0–20 μM	Inhibited VEGF and bFGF levels, decreased HIF-1α	Sui et al. (2017)
HUVECs	0–100 μM	Targeted HMGB1 release, reduced VCAM-1 and ICAM-1 secretion	Zhao et al. (2017)
HUVECs	0–30 μM	Modulated GLUT-1, activated HIF-1α pathway	Zhou et al. (2022)
Mice	10–20 mg/kg	Relieved VECs dysfunction, downregulated CLIC1	Zhu et al. (2017b)
HUVECs	50–200 ng	Inhibited VCAM-1 secretion and inflammatory cascade, suppressed NF-κB pathway	Liu et al. (2022)
Mice	10 mg/kg	Alleviated EndMT, inhibited tube formation reduction in VECs, regulated Akt/mTOR/p70S6K pathway	Jiang et al. (2019)
LDLR mice	15 mg/kg	Elevated macrophages efferocytosis, reduced lipid accumulation, inhibited foam cells formation	Wang et al. (2023)
Mice	10 mg/kg	Suppressing foam cell formation, regulated miR-214-3p/ATG16L1 axis and MAPK/mTOR-mediated autophagy	Qian et al. (2023)
Mice	30 mg/kg	Enhanced ATP-ABCA1 and ABCG1 expression, activated ERK/Nrf2/HO-1 pathway	Liu et al. (2014)
Mice	20 mg/kg	Promoted cholesterol efflux, alleviated lipid accumulation in macrophage, regulated Omentin-1/ABCA1 pathway	Tan et al. (2019)
Mice	10–90 mg/kg	Reduced the area of foam cells to the plaque, inhibited TLR4/MyD88/NF-κB pathway	Chen et al. (2019c)
Mice	30 mg/kg	Decreased LOX-1 expression, inhibited NF-κB pathway	Xu et al. (2012)
Rabbits	10 mg/kg	Promoted SR-BI and CE-TG interchange, regulated TG-rich lipoproteins pathway	Zhang et al. (2012)
Mice	10 mg/kg	Harmonized the crosstalk of autophagy and polarization in macrophages, activated KLF4 and suppressed miR-375	Chen et al. (2019b)
Mice	20 mg/kg	Downregulated COX-2, alleviated endothelial inflammation, modulated NF-κB pathway	Ma et al. (2023)
VSMCs	25–100 μmol/L	Suppressed TLR4/TAK1/NF-κB pathway	Meng et al. (2019)
Mice	60 mg/kg	Decreased IL-1β, IL-6, TNF-α and MMP-2, downregulated miR-146b and miR-155	Xuan et al. (2017)
Mice	10–20 mg/kg	Decreased ROS and MDA, downregulated CLIC1	Zhu et al. (2017b)
Macrophages	10–20 μM	Downregulated mRNA-33, IL-1β, IL-6 and TNF-α	Yang et al. (2019)
Mice	10 mg/kg	Mitigated inflammatory response, via activated TGF-β/PI3K/Akt/eNOS pathway	Wang et al. (2020c)
Macrophages	0–10 μM	Downregulated IL-1β, IL-6 and TNF-α, modulated miR-130b/Wnt5a	Yuan et al. (2020)
HUVECs	1–20 μM	Suppressed TNF-α-induced inflammatory response, downregulated VCAM-1, ICAM-1 and CX3CL1 expression	Chang et al. (2014)
Human blood	5 μM	Inhibited Aβ secretion in platelets, upregulated PI3K/Akt pathway	Shi et al. (2014)
Dendritic cells	0.01–10 μg/mL	Decreased CD86 and CD54, inhibited IL-1 and IL-12, restored endocytosis capacity	Li et al. (2014)
Mice	20 mg/kg	Attenuated NLRP3 inflammasome activation, decreased LOX-1 and CD36 expressions, ameliorated mitochondrial and lysosomal damage	Wen et al. (2020)
Mice	10 mg/kg	Decreased MMP-2 and MMP-9 expressions, suppressed spleen tyrosine kinase phosphorylation, inhibited NLRP3 inflammasome activation	Liu et al. (2024b)
Mice	20 mg/kg	Inactivated succinate dehydrogenase in macrophage, inhibited NLRP3 inflammasome activation	Liu et al. (2021)
Mice	60 mg/kg	Reduced NOX2, NOX4 and ROS, attenuated oxidative stress, down-regulating NF-κB pathway	Xuan et al. (2023)
Rabbits	3–30 mg/kg	Reduced ox-LDL production, elevated SOD and GSH-Px activities	Chen et al. (2012)
HUVECs	5–20 μM	Enhanced GSH-Px activity, activated PXR	Zhu et al. (2017a)
Rabbits	6.25–37.5 mg/kg	Decreased MDA level, CD40 expression and MMP-2 activity, increased SOD activity	Fang et al. (2008)
VECs	5–20 μg/μL	Relieved H_2_O_2_-induced VECs apoptosis, decreased MDA and ROS	Jia et al. (2012)
Human blood	5–100 μg/mL	Inhibited platelet activation, downregulated CD36, suppressed MKK4/JNK2 pathway	Wang et al. (2020a)
Mice	30 mg/kg	Elevated SOD level, activated ER, suppressed ERK pathway	Liu et al. (2015)
Rats	35–70 mg/kg	Relieved vessel lipid and calcium levels, protected against LDL oxidation	Tang et al. (2007)

### 4.2 Hyperlipidemia

Primary hyperlipidemia, a condition with significant genetic underpinnings, arises from either monogenic or polygenic defects affecting key components of lipoprotein metabolism, including receptors, enzymes, and apolipoproteins (Tomlinson, 2025). A prominent example is familial hypercholesterolemia, which typically results from genetic mutations affecting cell surface lipoprotein receptors, lipoprotein lipase functionality, or structural components of lipoproteins and apolipoproteins. These genetic alterations lead to congenital lipid metabolism disorders, ultimately manifesting as hyperlipidemia (Maidman et al., 2024). The pathogenesis of primary hyperlipidemia often involves complex gene-environment interactions, although the precise mechanisms remain incompletely understood. Genetic polymorphisms can significantly influence lipid metabolism pathways, while environmental factors may modulate gene expression and metabolic processes. Dietary habits play an essential role in the progression of hyperlipidemia, with excessive consumption of high-fat foods and caloric intake leading to fat accumulation that surpasses the body’s metabolic capacity. Furthermore, high carbohydrate intake can stimulate insulin secretion and enhance hepatic synthesis of very low-density lipoprotein, predisposing individuals to hypertriglyceridemia. Obesity represents a significant risk factor for hyperlipidemia, as increased adipose tissue mass disrupts lipid homeostasis and elevates circulating lipid levels. This condition often coexists with other metabolic disorders, including diabetes mellitus and hypertension, which can further exacerbate lipid metabolism abnormalities. Additionally, various systemic diseases can induce secondary hyperlipidemia through multiple mechanisms. Conditions such as diabetes mellitus, hypothyroidism, non-alcoholic fatty liver disease, nephrotic syndrome, and gout can interfere with normal lipid metabolic processes, leading to dyslipidemia (Driessen et al., 2023; Liu X. et al., 2024).

Recent studies have elucidated the multifaceted mechanisms through which Tan IIA and its derivatives modulate lipid metabolism and alleviate hyperlipidemia. Jia et al. demonstrated that Tan IIA ameliorates hyperlipidemia in rat models by modulating hepatic lipid metabolism through regulation of key enzymes and receptors, including cytochrome P450 family 7 subfamily A polypeptide 1 (CYP7A1), LDLR, sterol regulatory element binding protein-2 (SREBP-2), and lecithin cholesterol acyltransferase (LCAT), additionally, Tan IIA influenced macrophage cholesterol efflux through regulation of ABCA1 and CD36 expression (Jia L. Q. et al., 2016). Further mechanistic insights revealed that Tan IIA reduces lipid deposition by suppressing miR-33a and modulating the SREBP-2/proprotein convertase subtilisin/kexin type 9 (PCSK9) pathway (Jia L. et al., 2016). The therapeutic potential of STS has been demonstrated in various experimental models. Zhong et al. reported that STS protects against high-fat diet-induced hyperlipidemia by enhancing antioxidant capacity through activation of the Nrf2/HO-1 pathway (Zhong et al., 2020). Liu et al. further demonstrated that STS improves vascular dysfunction in hyperlipidemic mice by inhibiting spleen tyrosine kinase phosphorylation through modulation of the NLRP3 inflammasome-MMP2/9 pathway (Liu H. H. et al., 2024). At the cellular level, Tan IIA exhibits significant effects on lipid homeostasis and ER stress. Experimental evidence shows that Tan IIA reduces lipid accumulation by alleviating ER stress-induced unfolded protein response through activation of the peroxisome proliferator-activated receptor α (PPARα)/fibroblast growth factor 21 (FGF21) axis (Pi et al., 2024). In HepG2 cells, Tan IIA demonstrates protective effects against palmitate-induced lipid accumulation and apoptosis by suppressing ER stress markers, including glucose-regulated protein 78 (GRP78), activating transcription factor 6 (ATF6), and C/EBP homologous protein (CHOP) (Wang et al., 2020d). Furthermore, Gao et al. reported that Tan IIA attenuates hepatic lipid accumulation by down-regulating lipogenic genes such as fatty acid synthase (FASN), acetyl-CoA carboxylase-1 (ACC1), and stearoyl-CoA desaturase-1 (SCD1) through modulation of the liver X receptor α (LXRα)/SREBP1 pathway (Gao et al., 2021). The interplay between inflammation and lipid metabolism represents another therapeutic target of Tan IIA. Huang et al. demonstrated that Tan IIA reduces plasma lipid levels by ameliorating oxidative stress and inflammation through PPARγ activation and TLR4 downregulation (Huang et al., 2019). Similarly, Li et al. found that STS inhibits lipogenesis and moderates fat accumulation by suppressing pro-inflammatory cytokines through activation of the SIRT1/AMP-activated protein kinase α 1 (PRKAα1) pathway (Li X. X. et al., 2019).

### 4.3 Hypertension

Hypertension represents a systemic vascular disorder rather than a localized vascular lesion, involving multiple organ systems including the cerebral, coronary, renal, and retinal circulations (Mandorfer et al., 2025). The pathogenesis of hypertension involves complex interactions between vascular structural changes and neurohormonal regulation. Progressive vasoconstriction and luminal narrowing of resistance vessels, characterized by arterial wall thickening and reduced elasticity, lead to increased peripheral vascular resistance and subsequent elevation of blood pressure (BP). Besides, alterations in central nervous system function and dysregulation of neurotransmitter systems, including norepinephrine, dopamine, and enkephalins, can induce sympathetic nervous system hyperactivity. This results in elevated plasma catecholamine concentrations, which potentiate vasoconstriction of resistance arterioles and contribute to sustained BP elevation (Kim and Thiruvengadam, 2024). Additionally, activation of the renin-angiotensin-aldosterone system (RAAS) in response to various stimuli plays a pivotal role in the development and progression of hypertension. Renal pathophysiology significantly contributes to hypertension through mechanisms involving sodium and water retention, increased cardiac output, and subsequent elevation of peripheral vascular resistance. Furthermore, various modifiable risk factors have been identified in the etiology of hypertension, including chronic tobacco use, excessive dietary sodium intake, obesity, sleep deprivation, and chronic psychological stress (Faraci and Scheer, 2024). These factors interact with genetic predispositions to influence vascular tone regulation, fluid homeostasis, and neuroendocrine function, ultimately contributing to the development of sustained hypertension.

Pharmacological modulation of calcium (Ca^2+^) and large-conductance calcium-activated potassium (BKCa) channels represents a crucial mechanism for regulating vascular tone under both physiological and pathological conditions. STS has demonstrated significant hypotensive effects in experimental models, primarily through its dual action on BKCa channel activation and Ca^2+^ channel inhibition. The vasorelaxant properties of STS are mediated through multiple mechanisms, including BKCa channel activation, retardation of Ca^2+^ channels, and inhibition of Ca^2+^ influx in VSMCs (Zhang X. D. et al., 2018). At the molecular level, Tan et al. demonstrated that STS activates BKCa channels by enhancing the membrane expression of the α subunit in both HEK293 cells and VSMCs (Tan et al., 2014). This BKCa-dependent vasodilatory effect has been specifically observed in the mesenteric arteries of spontaneously hypertensive rats (Zhou et al., 2019). Furthermore, Yang et al. confirmed that STS activates high-conductance Ca^2+^-activated K^+^ channels in porcine coronary artery smooth muscle cells, providing additional evidence for its potassium channel-mediated vasodilatory effects (Yang et al., 2008). The vasodilatory mechanisms of Tan IIA extend beyond ion channel modulation. Wang et al. revealed that Tan IIA enhances eNOS expression in HUVECs through the transient receptor potential vanilloid 4 (TRPV4)-NO-protein kinase G (PKG) signaling pathway (Wang P. et al., 2024). This finding is complemented by Fan et al.'s observation that Tan IIA’s vasodilatory effects are modulated through ER-dependent eNOS activation and calcium mobilization (Fan et al., 2011). Interestingly, STS exhibits unique vascular effects in pregnancy-related conditions, demonstrating effective dilation of uterine arteries and direct vasodilatory effects on vascular resistance arteries in pregnant rats through NO-independent mechanisms (Morton et al., 2015). Additionally, Yu et al. demonstrated that Tan IIA exerts antihypertensive effects and inhibits cerebrovascular cell proliferation in hypertensive rats by attenuating ET-1 expression through phosphoinositide-dependent kinase 1 (PDK1) inactivation (Yu et al., 2015).

### 4.4 Myocardial injury and myocardial infarction (MI)

MI primarily results from the obstruction of coronary blood supply, leading to myocardial hypoxia and ischemia. The predominant etiology of MI is coronary atherosclerosis, which causes narrowing or complete occlusion of coronary arteries. This obstruction impedes adequate supply of oxygenated blood and essential nutrients to myocardium, resulting in ischemic injury. Prolonged or severe ischemia can induce metabolic disturbances and cardiomyocyte damage, ultimately leading to cellular necrosis, which represents a core pathological feature of MI (Zhai et al., 2025). The progression of MI involves distinct pathological phases. During the ischemic phase, cardiomyocytes remain viable but sustain significant damage. In the subsequent necrotic phase, these cells undergo complete functional loss, releasing intracellular enzymes and organelles that trigger inflammatory responses and tissue remodeling. Histopathological examination reveals characteristic features of necrotic cardiomyocytes, including cellular swelling, organelle disintegration, and nuclear degeneration (Wei et al., 2025). A major therapeutic challenge in MI is ischemia/reperfusion (I/R) injury, wherein the reintroduction of oxygen-rich blood to hypoxic cardiac tissue paradoxically exacerbates cellular damage through mechanisms involving calcium overload, as excessive extracellular calcium influx leads to intracellular calcium accumulation and increased cardiomyocyte death (Ghanta et al., 2025). The pathophysiology of MI also involves complex inflammatory and oxidative processes. Tissue damage triggers cell membrane degradation and increased production of arachidonic acid metabolites, which recruit leukocytes to the injured site. These inflammatory cells adhere to vascular endothelium, further exacerbating endothelial injury. Concurrently, an imbalance between ROS production and antioxidant defenses initiates lipid peroxidation cascades. Oxygen free radicals impair ATP production, induce inflammatory mediator release, and cause membrane damage, collectively contributing to cellular death and myocardial injury progression.

Chen et al. demonstrated that STS attenuates endotoxin-induced cardiomyocyte pyroptosis and autophagy in mice through inhibiting NLRP3 inflammasome activation (Chen P. et al., 2021). This finding is complemented by Hu et al.'s research showing that STS ameliorates ischemia-induced myocardial inflammation in canine models by modulating NLRP3 inflammasome activation through restoration of PPAR-α expression via the JAK2-STAT3 pathway (Hu et al., 2015). The therapeutic potential of Tan IIA extends to its interaction with IGF-2R, a critical mediator in myocardial injury pathogenesis. Experimental evidence shows that Tan IIA inhibits Ang II-induced apoptosis in rat H9c2 cardiomyocytes and prevents subsequent cardiac remodeling by suppressing β-catenin nuclear translocation and IGF-2R inactivation (Chen et al., 2017). Weng et al. further demonstrated that Tan IIA protects H9c2 cardiomyocytes from injury through IGF-2R inhibition mediated by PI3K/Akt pathway activation (Weng et al., 2015). The antioxidative properties of STS contribute significantly to its cardioprotective effects. Yan et al. reported that STS reduces myocardial apoptosis in murine models by mitigating oxidative stress through modulation of the Keap1-Nrf2 pathway (Yan et al., 2018). These findings collectively highlight the multifaceted mechanisms through which STS and Tan IIA exert their protective effects against myocardial injury and infarction.

MI initiates a cascade of pathological cardiac remodeling that often progresses to HF. Zhang et al. demonstrated that STS attenuates post-MI pathological remodeling in mice through multiple mechanisms, including reduction of myocardial necrosis, suppression of inflammatory responses, and promotion of angiogenesis (Zhang B. et al., 2022). Chai et al. further elucidated that Tan IIA inhibits cardiomyocyte pyroptosis in a rat HF model following acute MI by down-regulating key inflammatory mediators IL-1β, pro-IL-1β, NLRP3, and caspase-1 through suppression of the TLR4/NF-κB p65 pathway (Chai et al., 2023). Additionally, STS has shown cardioprotective effects in isoproterenol-induced MI models by modulating fatty acid β-oxidation (Wei et al., 2013). The ER, crucial for protein synthesis and processing in cardiomyocytes, plays a significant role in MI pathophysiology. Under conditions of hypoxia, nutrient deprivation, or calcium imbalance, ER stress response is activated. Prolonged ER stress can lead to dysfunction and subsequent cardiomyocyte damage. Tan IIA has been shown to mitigate cardiomyocyte injury by alleviating ER stress through upregulation of SIRT1 expression (Wu S. et al., 2023). The tumor suppressor phosphatase and tensin homolog (PTEN) has emerged as a key regulator of cardiomyocyte apoptosis. Wang et al. reported that Tan IIA improves cardiac function in MI mice by promoting angiogenesis through modulation of miR-499-5p and PTEN expression (Wang and Wu, 2022). This finding is supported by Zhang et al.'s demonstration that Tan IIA inhibits apoptosis in rat H9c2 cardiomyocytes by up-regulating miR-152-3p and down-regulating PTEN (Zhang et al., 2016). Recent advances in understanding post-MI cardiac repair have highlighted the critical role of macrophage reprogramming. Modulation of macrophage metabolic pathways and functional phenotypes has shown potential in promoting cardiac repair and improving cardiac function (Hu et al., 2024; Xie et al., 2024). Gao et al. demonstrated that Tan IIA facilitates cardiac repair in post-MI mice by reprogramming macrophage phenotypes through inactivation of the phosphoglycerate kinase 1 (PGK1)/pyruvate dehydrogenase kinase 1 (PDHK1) pathway and remodeling macrophage energy metabolism (Gao et al., 2024).

While reperfusion therapy remains the gold standard treatment for myocardial ischemia, it inevitably induces myocardial I/R injury (Zhu et al., 2023). A systematic meta-analysis revealed that Tan IIA exhibits significant cardioprotective effects in rat models of I/R injury at doses exceeding 5 mg/kg, primarily via suppression of oxidative stress (Zhang X. et al., 2024). The protective mechanisms of Tan IIA and its derivatives against I/R injury involve multiple molecular pathways. Zhong et al. demonstrated that Tan IIA alleviates cardiac microvascular I/R injury by reducing mitochondrial apoptosis through activation of the SIRT1-PGC1α pathway (Zhong et al., 2019). This finding is complemented by Long et al.'s research showing that STS attenuates microvascular I/R injury through inhibition of fibrinogen-like protein 2 (FGL2) expression, fibrin deposition, and inflammatory responses via mediating Akt and NF-κB pathways (Long et al., 2015). Li et al. further reported that Tan IIA ameliorates myocardial I/R injury by suppressing NLRP3 inflammasome activation and regulating Th17/Treg cells differentiation (Li et al., 2022). The antioxidative and anti-inflammatory properties of STS contribute to its cardioprotective effects, as evidenced by its ability to enhance HO-1 activity and mitigate I/R injury (Wei et al., 2014). Fang et al. demonstrated that Tan IIA improves myocardial ischemia by reducing cardiomyocyte apoptosis and modulating apoptotic markers caspase-3, Cyto c, and Apaf-1 in myocardial tissue (Fang et al., 2021). Moreover, the ataxia-telangiectasia mutated (ATM) kinase, a crucial regulator of DNA damage response and genomic stability, has emerged as a potential therapeutic target in myocardial injury (Kukreja, 2014). Sang et al. showed that Tan IIA protects against I/R injury in H9c2 cardiomyocytes via ATM-mediated activation of the GADD45/ORC pathway (Sang et al., 2024). Besides, the composition of extracellular matrix changes significantly, collagen secretion decreases while hyaluronic acid accumulation increases competitively during acute MI, activated cardiac fibroblasts produce hyaluronic acid through hyaluronan synthase 2 (HAS2) (Little et al., 2025). Tan IIA has been shown to mitigate I/R injury in human AC16 cardiac cells by targeting the HAS2/fibroblast growth factor 9 (FGF9) axis, thereby reducing inflammation and oxidative stress (Wang Y. et al., 2024). Furthermore, Tan IIA protects against mitochondrial dysfunction through modulation of voltage-dependent anion channel 1 (VDAC1), an essential regulator of mitochondrial-cytoplasmic exchange. Hu et al. demonstrated that Tan IIA inhibits ferroptosis and apoptosis in H9c2 cardiomyocytes by down-regulating VDAC1 and preventing oxidative stress (Hu T. et al., 2023). The cardioprotective effects of Tan IIA extend to mitochondrial preservation. It protects H9c2 cardiomyocytes against anoxia/reoxygenation injury by inhibiting mPTP opening and apoptosis through upregulation of 14-3-3η, a protein that played a momentous protective role in cardiomyocytes (Zhang Z. et al., 2018). Wen et al. further demonstrated that Tan IIA alleviates I/R injury by regulating autophagy and maintaining mitochondrial function through 14-3-3η-mediated modulation of the Akt/Beclin1 pathway (Wen et al., 2023). Emerging therapeutic strategies utilizing mesenchymal stem cells (MSCs) have shown promise in CVDs treatment. Li et al. found that exosomes derived from Tan IIA-pretreated MSCs exert cardioprotective effects in myocardial I/R models by up-regulating miR-223-5p and inactivating CCR2, a protein-coding gene highly expressed in cardiomyocytes (Li S. et al., 2023).

**FIGURE 3:**
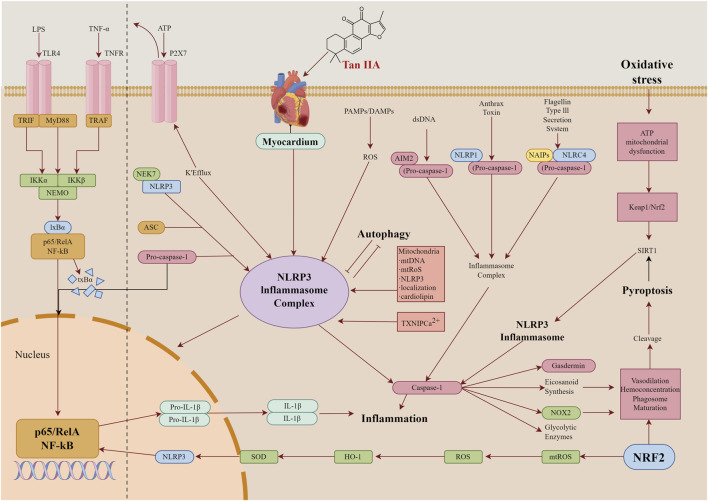
Tan IIA mediate cross-talk among NLRP3 inflammasome, NF-κB, and Nrf2 pathways in myocardial protection.

Interestingly, Tan IIA demonstrates significant myocardial protective effects by modulating the crosstalk between the NLRP3 inflammasome and the Nrf2 antioxidant pathway, with its efficacy exhibiting a clear dose-dependent pattern. Mechanistically, Nrf2 activation is more responsive to oxidative stress, whereas NLRP3 inhibition requires higher Tan IIA concentrations to effectively suppress the inflammatory signaling cascade. At low doses, Tan IIA preferentially enhances antioxidant defenses, markedly up-regulating SOD activity and reducing MDA levels, while exerting only a modest inhibitory effect on NLRP3 inflammasome activation (Chen P. et al., 2021). In contrast, medium-dose Tan IIA achieves a balanced modulation of both pathways, simultaneously reducing myeloperoxidase (MPO) activity and IL-1β levels while significantly restoring ATP content, indicating coordinated anti-inflammatory and antioxidant actions (Vidal-Gomez et al., 2025). However, at high doses, Tan IIA may excessively suppress inflammatory responses, potentially compromising immune defense mechanisms (Wu S. et al., 2023). Collectively, Tan IIA mediates cardioprotection through a dynamic Nrf2-NLRP3 interaction network, exhibiting dose-dependent preferential effects: low-dose regimens favor anti-oxidative activity, high-dose treatments enhance anti-inflammatory efficacy, and intermediate doses promote synergistic regulation. These findings provide a mechanistic rationale for optimizing Tan IIA dosing in clinical applications. Future studies should further elucidate tissue-specific dose-response relationships and evaluate long-term safety profiles to facilitate translational development. A comprehensive summary of the myocardial protective effect of Tan IIA through crosstalk among NLRP3 inflammasome, NF-κB, and Nrf2 pathways is presented in Figure 3.

### 4.5 Cardiac hypertrophy, fibrosis, and heart failure (HF)

Cardiac hypertrophy, characterized by an increase in myocardial cells volume and consequent thickening of the ventricular wall, represents a complex adaptive response that can be either physiological or pathological in nature. Physiological hypertrophy, exemplified by the athlete’s heart, contrasts with pathological hypertrophy commonly observed in conditions such as hypertension and valvular heart disease. The pathogenesis of pathological myocardial hypertrophy primarily involves activation of neuroendocrine systems, particularly the RAAS and sympathetic nervous system, along with dysregulation of cellular signaling pathways including calcium ion channels and MAPK cascades (Chen et al., 2025). Myocardial fibrosis, characterized by excessive deposition of collagen and extracellular matrix components, represents another critical pathological process in cardiac remodeling. This fibrotic transformation, frequently associated with aging, obesity, and diabetes mellitus, significantly impairs cardiac function through multiple mechanisms. The accumulation of fibrous tissue increases myocardial stiffness, compromising ventricular diastolic function and impairing proper cardiac filling, ultimately reducing stroke volume and cardiac output (Skorka et al., 2025; Zhang et al., 2023). Furthermore, myocardial fibrosis disrupts the typical cellular architecture and electrophysiological properties of cardiac tissue, creating a substrate for arrhythmias that further compromise cardiac efficiency and contribute to the progression of HF (Lee et al., 2025). The excessive extracellular matrix deposition also physically restricts cardiomyocyte contractility, reducing myocardial contractile force and exacerbating cardiac dysfunction (Hosseinzadeh et al., 2024). The interplay between myocardial hypertrophy and fibrosis creates a vicious cycle in HF pathophysiology. Hypertrophic changes increase cardiac wall stress, promoting further cardiomyocyte enlargement and subsequent fibrotic transformation. The resulting fibrosis exacerbates myocardial stiffness and dysfunction, creating a self-perpetuating cycle of cardiac remodeling and functional deterioration. This complex interaction between structural and functional alterations underlies the progressive nature of HF development.

Tan IIA has demonstrated significant cardioprotective effects against cardiac hypertrophy and fibrosis through multiple molecular mechanisms. Experimental studies have revealed that Tan IIA attenuates cardiac hypertrophy in rat models by down-regulating collagen type I α-1 (COL1A1) mRNA expression through inhibition of the cystatin C (Cys-C)/Wnt pathway (Feng et al., 2017). Jiang et al. further elucidated Tan IIA’s potent anti-fibrotic properties, which are mediated through modulation of both TGF-β/Smad and MAPK/ERK pathways (Jiang et al., 2024). The therapeutic potential of Tan IIA extends to Ang II-induced cardiac fibrosis, where it enhances NO production and eNOS phosphorylation while inhibiting ERK phosphorylation (Chan et al., 2011). Zhang et al. demonstrated that Tan IIA alleviates acid-induced fibrosis by targeting the Nrf2-NLRP3 pathway, thereby reducing oxidative stress and inflammatory responses (Zhang W. et al., 2024). In the context of hypertension-induced cardiac remodeling, Tan IIA exhibits multifaceted protective effects. It mitigates left ventricular myocardial changes by reducing MMP-9 and tissue inhibitor of metalloproteinase-1 (TIMP-1) expression, thereby improving cardiac function in renovascular hypertensive rats (Fang et al., 2010). Pang et al. reported that Tan IIA inhibits hypertension-induced left ventricular hypertrophy and fibrosis by modulating the TGF-β/Smads pathway while reducing MDA levels and enhancing SOD activity (Pang et al., 2014). Additionally, Jiang et al. found that Tan IIA suppresses cardiomyocyte hypertrophy and apoptosis in spontaneously hypertensive rats through regulation of Bcl-2, Bax, and p53 expression (Jiang et al., 2013). The antioxidative properties of Tan IIA contribute significantly to its cardioprotective effects. It improves cardiac function and reduces fibrosis in hypertensive models by decreasing NADPH oxidase activity, a major source of ROS in the heart (Wang et al., 2011). This mechanism is further supported by studies showing Tan IIA’s ability to inhibit H_2_O_2_-stimulated cardiac fibroblasts through suppression of collagen synthesis and NADPH oxidase activity (Wang et al., 2013). Tan IIA’s anti-hypertrophic effects are also mediated through inflammation modulation. It attenuates transverse aortic constriction-induced cardiac hypertrophy by reducing IL-6 and TNF-α levels, decreasing MDA content, and enhancing SOD activity via SIRT1 activation (Feng et al., 2016). Furthermore, Zhang et al. demonstrated that Tan IIA inhibits galectin-3 expression through modulation of N6-methyladenosine (m6A) methylation, thereby attenuating cardiac hypertrophy (Zhang M. et al., 2022). At the molecular level, Tan IIA influences microRNA regulation of cardiac remodeling. It inhibits hypertrophy and collagen deposition in rat heart tissues through upregulation of miR-618 (Yan et al., 2022). Mao et al. further reported that Tan IIA reduces collagen deposition in human cardiac fibroblasts by modulating MMP-2 and MMP-9 expression and regulating elastin deposition (Mao et al., 2014). The cardioprotective effects of Tan IIA extend to adrenergic stress-induced cardiac remodeling. It mitigates isoproterenol-induced cardiac hypertrophy by reducing brain natriuretic peptide (BNP), atrial natriuretic peptide (ANP), and β-myosin heavy chain (β-MHC) levels through suppression of the calcineurin/NFATc3 pathway (Tan et al., 2011).

Emerging evidence has highlighted the crucial role of gut microbiota dysbiosis and gut-brain axis dysfunction in the pathogenesis of HF. Zhu et al. demonstrated that Tan IIA significantly attenuates myocardial apoptosis and fibrosis, subsequently improving cardiac hypertrophy and function in mice. This cardioprotective effect is mediated through inhibition of inflammatory responses and restoration of intestinal barrier integrity via modulation of the gut-brain axis (Zhu et al., 2024). The therapeutic potential of Tan IIA extends to chemotherapy-induced cardiotoxicity. Xu et al. reported that Tan IIA ameliorates doxorubicin-induced myocardial structural alterations and myofibrillar damage in HF mice through activation of the ERK1/2 signaling pathway (Xu et al., 2022). Wang et al. further elucidated that Tan IIA mitigates doxorubicin-induced HF by restoring autophagic flux through regulation of the Beclin1/lysosomal-associated membrane protein-1 (LAMP1) pathway (Wang et al., 2019). Innovative drug delivery systems have been developed to enhance Tan IIA’s therapeutic efficacy. Zhao et al. designed ROS-responsive triphenylphosphine-modified Tan IIA micelles, which demonstrated superior cardioprotective effects in doxorubicin-induced HF by reducing oxidative stress and inflammatory cytokine infiltration through mitochondrial activation in cardiomyocytes (Zhao et al., 2025). The molecular mechanisms underlying Tan IIA’s cardioprotective effects involve multiple signaling pathways. Experimental evidence shows that Tan IIA protects against HF by inhibiting myocardial apoptosis and promoting autophagy through activation of the AMPK-mTOR signaling cascade (Zhang et al., 2019). Chen et al. further demonstrated that Tan IIA attenuates cardiac dysfunction and fibrosis in HF models by reducing TGF-β, α-SMA, and MMP-9 expression in cardiac fibroblasts through suppression of oxidative stress (Chen R. et al., 2021). In the context of pressure overload-induced HF, Tan IIA exhibits significant therapeutic potential. Chronic pressure overload, characterized by increased resistance during cardiac contraction and relaxation, leads to pathological cardiomyocyte hypertrophy and subsequent cardiac insufficiency. Li et al. demonstrated that Tan IIA alleviates ventricular remodeling and improves cardiac function in HF mice via restraining inflammatory responses and cardiomyocyte apoptosis (Li X. et al., 2019).

### 4.6 Arrhythmia

Arrhythmia is defined as a disturbance in the frequency and/or rhythm of cardiac contractions resulting from abnormalities in the initiation and/or propagation of electrical impulses within the heart. The underlying mechanisms primarily involve irregularities in impulse formation and conduction, which can be influenced by various pathological factors, including pharmacological effects, drug toxicity, electrolyte imbalances, anesthesia, surgical interventions, cardiac catheterization, and autonomic nervous system dysfunction (Mondejar-Parreno et al., 2025). Arrhythmia arises when the electrical signals responsible for regulating cardiac contractions are delayed or obstructed. This disruption can occur due to either malfunctioning specialized cardiac pacemaker cells or impaired transmission of electrical impulses through the heart’s conduction system (Zeitler et al., 2024). Additionally, arrhythmia may result from ectopic electrical activity originating in other regions of the heart or abnormal propagation of impulses from pacemaker cells, both of which can interfere with the heart’s regular rhythmic activity. Furthermore, insufficient coronary blood flow can compromise the metabolic and functional integrity of cardiomyocytes, leading to alterations in myocardial electrophysiological properties and subsequent arrhythmogenesis (Yang et al., 2025). Electrolytes play a critical role in maintaining the regular electrophysiological activity of cardiomyocytes. Imbalances in the concentrations of key ions such as potassium, sodium, and calcium can significantly affect cardiomyocyte excitability, automaticity, and conductivity, thereby predisposing to arrhythmia.

**FIGURE 4:**
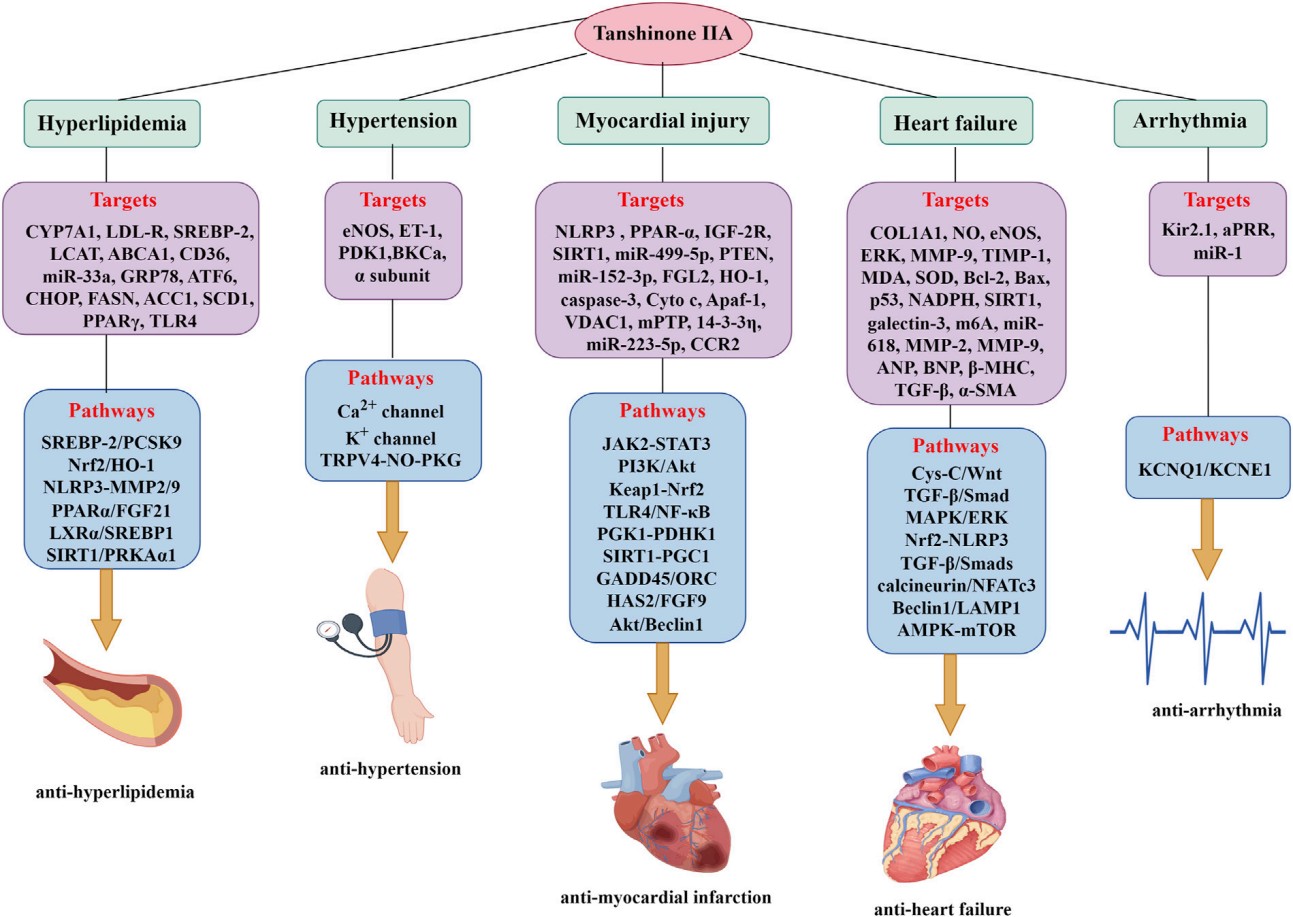
The molecular targets and mechanisms regulated by Tan IIA in treating CVDs.

He et al. demonstrated that Tan IIA effectively ameliorates atrial fibrillation in rabbits, with its anti-arrhythmic properties primarily attributed to the modulation of atrial electrophysiology. Specifically, Tan IIA prolongs atrial postrepolarization refractoriness (aPRR) and moderately increases interatrial conduction time (He et al., 2016). Shan et al. further elucidated that Tan IIA reduces the duration of arrhythmia and lowers the incidence of ventricular tachycardia (VT) and ventricular fibrillation (VF) in rats. This effect is mediated through the upregulation of Kir2.1 expression by inhibiting miR-1, which expressed in the heart that regulates various ion channels and myocardial electrical activity under pathological conditions (Shan et al., 2009). Sun et al. identified Tan IIA as a potential activator of the human cardiac slow delayed-rectifier K^+^ current (*I*_Ks_). Their findings revealed that Tan IIA activates human cardiac KCNQ1/KCNE1 potassium channels in HEK 293 cells by altering the channels’ kinetic properties (Sun et al., 2008). Despite these promising findings, research on the anti-arrhythmic effects of Tan IIA remains limited, and the precise pharmacological mechanisms warrant further investigation. A comprehensive summary of the effects and molecular targets regulated by Tan IIA in treating CVDs is presented in Figure 4 and Table 2. A comprehensive summary of Tan IIA’s efficacy against conventional cardiovascular drugs for CVDs is presented in Table 3.

**TABLE 2:** The cardiovascular protective mechanisms of Tan IIA.

Diseases	Animals or cells	Range of dosage	The specifific molecular mechanisms	Ref.
Hyperlipidemia	Rats	10 mg/kg	Affected HDL subfractions distribution, regulated CYP7A1, LDL-R, SREBP-2 and LCAT in liver, ABCA1 and CD36 in macrophage	Jia et al. (2016b)
	Rats	10 mg/kg	Suppressed miR-33a and ABCA1, modulated SREBP-2/PCSK9 pathway	Jia et al. (2016a)
	Rats	10 μg/kg	Elevated antioxidant capacity, activated Nrf2/HO-1 pathway	Zhong et al. (2020)
	Mice	10 mg/kg	Mitigated dysvasodilation induced by hyperlipidemia, modulated NLRP3 inflammasome-MMP2/9 pathway	Liu et al. (2024b)
	Mice	10–20 mg/kg	Alleviated endoplasmic reticulum stress-induced unfolded protein response, activated PPARα/FGF21 axis	Pi et al. (2024)
	HepG2 cells	10 μM	Suppressed GRP78, ATF6, and CHOP, inhibited endoplasmic reticulum stress	Wang et al. (2020d)
	HepG2 cells	1–10 μM	Attenuated lipogenic gene FASN, ACC1, and SCD1, regulating LXRα/SREBP1 pathway	Gao et al. (2021)
	Rats	10 mg/kg	Ameliorated oxidative stress and inflammatory response, stimulated PPARγ and downregulated TLR4	Huang et al. (2019)
	Mice	10–20 mg/kg	Suppressed TNF-α, TGF-β and IL-1β, activated SIRT1/PRKAα1 pathway	Li et al. (2019b)
Hypertension	Rats	20–200 μM	Activated BKCa channel, blocked Ca^2+^ channel, inhibited Ca^2+^ influx to VSMCs	Zhang et al. (2018a)
	HEK293 cells and VSMCs	40–160 μM	Enhanced membranous level of α subunit, activated BKCa channel	Tan et al. (2014)
	Porcine coronary artery smooth muscle cells	20–150 μM	Activated high conductance Ca^2+^-activated K^+^ channels	Yang et al. (2008)
	HUVECs	1 μM	Increased eNOS expression, mediated TRPV4-NO-PKG pathway	Wang et al. (2024a)
	VECs	0–10 μM	Activated eNOS, mobilized calcium	Fan et al. (2011)
	Rats	0.05–5 mg/kg	Attenuated ET-1, inactivated PDK1	Yu et al. (2015)
Myocardial infarction	Mice	10 mg/kg	Mitigated cardiomyocyte pyroptosis and autophagy, inhibited NLRP3 inflammasome activation	Chen et al. (2021a)
	Dogs	1.3–5.2 mg/kg	Inhibited cardiac NLRP3 inflammasome activation, restored PPAR-α, regulated JAK2-STAT3 pathway	Hu et al. (2015)
	Rat H9c2 cardiomyocytes	40 μM	Suppressed β-catenin nuclear translocation, inactivating IGF-2R	Chen et al. (2017)
	Rat H9c2 cardiomyocytes	0–100 μM	Inhibited IGF-2R, activated PI3K/Akt pathway	Weng et al. (2015)
	Mice	10 mg/kg	Alleviated myocardial apoptosis, inhibited oxidative stress damage, targeted Keap1-Nrf2 pathway	Yan et al. (2018)
	Mice	20.8 mg/kg	Decreased myocardial necrosis, inhibited inflammation, and elevated angiogenesis	Zhang et al. (2022a)
	Rats	1.5 mg/kg	Downregulated IL-1β, pro-IL-1β, NLRP3, caspase-1, suppressed TLR4/NF-κB p65 pathway	Chai et al. (2023)
	Rats	4–16 mg/kg	Inhibited fatty acid β-oxidation	Wei et al. (2013)
	Mice	10–50 mg/kg	Suppressed endoplasmic reticulum stress, upregulated SIRT1	Wu et al. (2023b)
	Mice	50 mg/kg	Induced angiogenesis, upregulated miR-499-5p, downregulated PTEN	Wang and Wu (2022)
	Rat H9c2 cardiomyocytes	1–10 μg/mL	Increased miR-152-3p, downregulated PTEN	Zhang et al. (2016)
	Mice	20 mg/kg	Reprogrammed macrophages phenotype, inactivated PGK1-PDHK1 pathway, reshaped macrophages energy metabolism mode	Gao et al. (2024)
Myocardial ischemia/reperfusion injury	Mice	5–25 mg/kg	Reduced mitochondrial apoptosis, activated SIRT1-PGC1α pathway	Zhong et al. (2019)
	Rats	4–8 mg/kg	Inhibited FGL2 expression, fibrin deposition and inflammatory response, mediated Akt and NF-κB pathways	Long et al. (2015)
	Rats	15 mg/kg	Inhibited NLRP3 inflammasome activation, suppressed Th17/Treg cells differentiation	Li et al. (2022)
	Rats	8 mg/kg	Inhibited oxidative stress and inflammatory response, increased HO-1 activity	Wei et al. (2014)
	Rats	10 mg/kg	Decreased myocardiocytes apoptosis, reduced caspase-3, Cyto c and Apaf-1	Fang et al. (2021)
	Rat H9c2 cardiomyocytes	10 μM	Activated GADD45/ORC pathway, upregulated ATM	Sang et al. (2024)
	Human AC16 cardiac cells	0–60 μM	Inhibited inflammation and oxidative stress damage, targeted HAS2/FGF9 axis	Wang et al. (2024c)
	Rat H9c2 cardiomyocytes	1–32 μM	Inhibited oxidative stress damage, downregulated VDAC1	Hu et al. (2023b)
	Rat H9c2 cardiomyocytes	2–32 μM	Prevented mPTP opening and apoptosis, upregulated 14-3-3η	Zhang et al. (2018b)
	Rats	20 mg/kg	Inhibited excessive autophagy, maintained mitochondrial function, upregulated 14-3-3η, regulated Akt/Beclin1 pathway	Wen et al. (2023)
	Rats	75 μg	Upregulated miR-223-5p, inactivated CCR2	Li et al. (2023a)
Cardiac hypertrophy and fibrosis	Rats	1–10 mg/kg	Decreased COL1A1 mRNA, suppressed Cys-C/Wnt pathway	Feng et al. (2017)
	Mice	10 mg/kg	Modulated TGF-β/Smad and MAPK/ERK pathways, attenuated fibrosis	Jiang et al. (2024)
	Cardiac fibroblast	0–10 μM	Attenuated cardiac fibrosis, increased NO and eNOS phosphorylation, inhibited ERK phosphorylation	Chan et al. (2011)
	HK-2 cells	1–100 μM	Suppressed oxidative stress and inflammatory response, regulated Nrf2-NLRP3 pathway	Zhang et al. (2024a)
	Rats	35–70 mg/kg	Inhibited collagen metabolism, decreased MMP-9 andTIMP-1	Fang et al. (2010)
	Rats	5–15 mg/kg	Decreased MDA contents and increased SOD activities, modulated TGF-β/Smads pathway	Pang et al. (2014)
	Rats	10 mg/kg	Upregulated Bcl-2, downregulated Bax and p53	Jiang et al. (2013)
	Rats	35–70 mg/kg	Decreased NADPH oxidase activity	Wang et al. (2011)
	Cardiac fibroblasts	0.1–10 μM	Blocked collagen synthesis, decreased NADPH oxidase activity	Wang et al. (2013)
	Rats	15 mg/kg	Decreasing IL-6, TNF-α levels and MDA content, and elevating SOD activity through activating SIRT1	Feng et al. (2016)
	Mice	10 mg/kg	Suppressed galectin-3, modulated m6A methylation	Zhang et al. (2022b)
	Rats	10 mg/kg	Inhibited collagen deposition, elevated miR-618	Yan et al. (2022)
	Human cardiac fibroblasts	0.1–10 μM	Downregulated MMP-2 and MMP-9, modulated elastin ultimate net deposition	Mao et al. (2014)
	Rat ventricular myocytes	10–100 μM	Decreased ANP, BNP and β-MHC, suppressed calcineurin/NFATc3 pathway	Tan et al. (2011)
Heart failure	Mice	30 mg/kg	Suppressed myocardial apoptosis and fibrosis, alleviated hypertrophy, inhibited inflammatory reaction, targeted gut-brain axis	Zhu et al. (2024)
	Mice	2.5–10 mg/kg	Activated ERK1/2 pathway	Xu et al. (2022)
	Mice	10 mg/kg	Restored autophagosome/autolysosome balance, regulated Beclin1/LAMP1 pathway	Wang et al. (2019)
	Mice	1.5 mg/kg	Reduced oxidative stress damage and inflammatory cytokine infiltration, activating cardiomyocyte mitochondria	Zhao et al. (2025)
	Rats	1.5 mg/kg	Suppressed myocardial apoptosis and induced autophagy, activated AMPK-mTOR pathway	Zhang et al. (2019)
	Rats	1.5 mg/kg	Attenuated cardiac dysfunction and fibrosis, decreased TGF-β, α-SMA, and MMP-9 in cardiac fibroblasts, suppressed oxidative stress	Chen et al. (2021b)
	Rats	5–20 mg/kg	Suppressed inflammatory reaction and cardiomyocytes apoptosis	Li et al. (2019a)
Arrhythmia	Rabbits	10 μM	Elevated aPRR, increased interatrial conduction time	He et al. (2016)
	Mice	10 mg/kg	Increased Kir2.1, inhibited miR-1	Shan et al. (2009)
	HEK 293 cells	10–100 μM	Activated cardiac KCNQ1/KCNE1 potassium channels, affected the channels’ kinetics	Sun et al. (2008)

**TABLE 3:** Comparative efficacy of Tan IIA vs. conventional cardiovascular drugs.

Category	Tanshinone IIA/STS	Statins	Angiotensin-converting enzyme (ACE) inhibitors	Nitrate medications
Primary Mechanism	Multi-target effects: calcium channel antagonism, antioxidation, anti-inflammation, promoting angiogenesis, anti-platelet aggregation, and anti-fibrosis	Inhibit HMG-CoA reductase, reduce cholesterol synthesis, lower LDL-C, and stabilize atherosclerotic plaques	Inhibit ACE, reduce Ang II production, lower blood pressure, and reverse myocardial hypertrophy and fibrosis	Dilate blood vessels through the NO-cGMP pathway to relieve angina pectoris
Advantages of special groups	Suitable for the elderly and patients with impaired liver and kidney functions	Dosage needs to be adjusted (due to liver and kidney dysfunction)	Contraindicated for pregnancy and bilateral renal artery stenosis	Contraindicated for severe hypotension
Common adverse reactions	Mild allergic reactions	Myalgia, hepatotoxicity, diabetes risk	Cough, hyperkalemia, hypotension	Headache, hypotension, tolerance
Combination Potential	Synergistic with statins/anti-platelets	Combined with ezetimibe/PCSK9 inhibitors	Combined with ARBs/β-blockers	Combined with β-blockers/calcium channel blockers (CCBs)

## 5 Conclusion and future perspective

In conclusion, Tan IIA represents a valuable natural compound with immense potential in cardiovascular medicine. Its multifaceted pharmacological properties, including potent anti-inflammatory, antioxidant, and anti-atherosclerotic effects, position it as a compelling candidate for managing a spectrum of CVDs such as atherosclerosis, acute myocardial infarction, and coronary heart disease. Recent advancements have elucidated Tan IIA’s ability to modulate key signaling pathways implicated in cardiovascular pathology, including the inhibition of pro-inflammatory cytokine expression, reduction of oxidative stress, and stabilization of atherosclerotic plaques. By targeting multiple pathological processes simultaneously, Tan IIA demonstrates remarkable versatility and therapeutic efficacy. As research continues to evolve, Tan IIA holds the potential to revolutionize cardiovascular treatment paradigms, offering innovative, integrative therapeutic strategies that could significantly improve clinical outcomes for patients with CVDs. Nevertheless, the potential adverse effects associated with prolonged and/or high-dose administration of Tan IIA warrant careful consideration and should not be overlooked. Some individuals may exhibit allergic reactions to Tan IIA, manifesting as pruritus, erythema, swelling, and cutaneous eruptions following administration. In severe cases, these hypersensitivity reactions may be accompanied by respiratory distress. Tan IIA may also exert irritative effects on the gastrointestinal mucosa, potentially inducing epigastric pain, nausea, emesis, abdominal discomfort, and diarrhea. Prolonged administration on an empty stomach may result in gastric mucosal injury. Due to its pharmacologic properties of promoting blood circulation, Tan IIA administration may lead to accelerated systemic circulation, potentially causing facial flushing in some patients. Furthermore, it may interfere with the coagulation cascade, potentially resulting in coagulation disorders. This may present as cutaneous ecchymosis, ocular hemorrhage, hematuria, menorrhagia, or gastrointestinal bleeding. In severe instances, it may precipitate critical hemorrhagic events, including but not limited to cerebral hemorrhage. Upon the occurrence of the aforementioned adverse effects, immediate discontinuation of Tan IIA is warranted, followed by appropriate symptomatic management.

Despite its considerable promise, the clinical application of Tan IIA faces several challenges. Key limitations include its low bioavailability, insufficient clinical data, and an incomplete understanding of its therapeutic mechanisms and molecular targets. To date, most studies have been confined to animal models or small-scale clinical trials, underscoring the need for large-scale, high-quality clinical studies to validate its safety and efficacy. Moving forward, interdisciplinary collaboration and innovative research methodologies will be critical to unlocking the full therapeutic potential of Tan IIA. Future research should prioritize optimizing drug delivery systems, elucidating multi-target synergistic mechanisms, and developing highly effective derivative which not only improves the bioavailability of Tan IIA, but also enhances its pharmacological activity and reduces its adverse effects. The development of new derivatives and compounds containing Tan IIA such as Compound Danshen Dripping Pills is of great significance and is expected to provide a new option for treating CVDs (Wang R. et al., 2024). These efforts will be essential to bridge the gap between traditional Chinese medicine and modern therapeutics, ultimately facilitating the transformation of Tan IIA into a widely adopted clinical treatment for CVDs.
